# Psychosocial Challenges Associated with Caregiving in the Context of Pediatric HIV in Rural Eastern Cape

**DOI:** 10.3389/fpubh.2017.00127

**Published:** 2017-06-12

**Authors:** Antonio G. Lentoor

**Affiliations:** ^1^Department of Psychiatry and Mental Health, Valkenberg Hospital, University of Cape Town, Cape Town, South Africa

**Keywords:** psychosocial, human immunodeficiency virus/acquired immunodeficiency syndrome, pediatric, caregiving, low-resource setting

## Abstract

**Background:**

While survival among human immunodeficiency virus (HIV)-infected children has increased due to combination antiretroviral therapy, many children remain vulnerable to the adverse effects of poverty and family disruptions as a result of the loss of one or both biological parents to acquired immunodeficiency syndrome. The aim of this qualitative study was to develop an understanding of the psychosocial challenges experienced by caregivers caring for a child with perinatally acquired HIV.

**Method:**

A series of interviews were conducted with 44 HIV-positive and -negative primary caregivers of HIV+ children. Data were analyzed through interpretative phenomenological analysis using NVivo8 software.

**Findings:**

The findings suggest that caregiving is compromised by inadequate, financial resources and single-headed households where mainly grandparents assume the role of primary caregivers of HIV+ children. HIV remains a stigmatized illness that weakens support networks, as well as timeous and free accessibility to healthcare. This has a negative impact on the mental health of caregivers, with the majority of women in the study displaying symptoms of depression.

**Conclusion:**

The findings highlight the contextual challenges of caregiving in the presence of HIV, which impacts negatively on social ecology of the families. The need for interventions to enhance resilience and coping in families confronted with HIV is indicated.

## Introduction

The human immunodeficiency virus (HIV) and acquired immunodeficiency syndrome (AIDS) continue to pose a global health threat (1), with the AIDS pandemic having claimed millions of lives in the world over since it was first identified in the early 1980s (1, 2). To date, it remains an incurable disease and millions continue to suffer and even die from it annually, particularly in low-resource settings (3). The devastating impact of HIV/AIDS on children, adolescents, caregivers, and families remain a major challenge as HIV/AIDS presents numerous unique and complex medical, financial, and psychosocial challenges to families (1). Globally, women of childbearing age are disproportionately more affected by AIDS, and a consequence of this is that a significant number of children are in turn infected with HIV perinatally (1, 2, 4). Women are often confronted with the double burden of being infected and having the responsibility of caring for their HIV-positive children (5, 6). Women 15 years and older constituted 3.4 million of the total number of adults living with HIV in 2012, while new HIV infections among children (0–14 years) was estimated at around 21,000 (95% CI: 19,000–32,000) in 2012 (6). In the case of perinatally HIV (PHIV)-infected children from poor resource African countries whose biological parents have died, mainly grandmothers and aunts have assumed a caregiver role (7–9). It has been shown that South Africa has more than 60% of double orphans being raised by a grandmother or relative other than the biological parent (10). These children and caregivers are also often living in poverty which poses additional challenges. It is argued that poverty exposes PHIV-positive children to various psychosocial risk factors, which often co-occur and impact negatively on the child’s neurocognitive development, thereby contributing to poor health, lack of readiness for school, poor academic performance, and psycho-emotional problems (11). The largest burden of the HIV pandemic, combined with poverty, remains in low- and middle-income countries and results in various social and economic challenges for already vulnerable group of people (12). Therefore, the aim of this qualitative study was to develop an understanding of the psychosocial challenges experienced by caregivers caring for a child with perinatally acquired HIV from a resource-limited rural community in the Eastern Cape, South Africa.

## Methodology

### Setting and Sampling

The research was conducted within the Buffalo City Metropolitan Municipality, Eastern Cape, South Africa. The Eastern Cape is also one of the poorest provinces in South Africa, with high levels of under development and an unemployment rate of about 24.3% (13). A sample of 44 biological and non-biological caregivers of children infected with HIV perinatally and treated on antiretroviral therapy was conveniently recruited from the pediatric department from a local hospital. Only caregivers 18 years and older, whose children were HIV+ and were in the age range of 31.38–92.78 months (*M* = 63.13), were included in the study.

### Procedure and Data Collection

All the participants were approached during their child’s routine visit to the HIV clinic in the Pediatric Department after formal access was granted by the Head of Department. The research was explained to all the caregivers in English and isiXhosa by a clinical psychologist and an isiXhosa-speaking clinical social worker who had prior experience of working with families affected by HIV/AIDS. All the interviews with the caregivers were conducted in the hospital at the time of visitation after formal written informed consent was obtained from caregivers.

## Ethical Consideration

The University of KwaZulu Natal’s Biomedical Research Ethics Committee (Protocol Number: BE252/11) and Ethics Committee of East London Hospital Complex approved the study. Each caregiver was informed about the nature of the study and was reassured that their participation or otherwise would not affect the management and quality of care of their children at the hospital. All the caregivers who understood and agreed to participate in the study gave written informed consent. All the participants were informed of the availability of psychological and social welfare services should the need for this arise.

## Measure

An open semi-structured interview schedule (SIS) was used to obtain the qualitative data from the participants. The choice of using a SIS was informed through the theoretical underpinnings of interpretative phenomenological analysis (IPA) (14), which allowed the researcher to explore and gain a better understanding of the experiences encountered by the participants of a particular psychosocial and health phenomenon. As part of phenomenological psychology, the goal of the study was to: (i) generate and collect data regarding the experiences of caregivers caring for HIV-positive children; (ii) identify key themes from caregivers’ experiences with respect to caring for an HIV-positive child; and (iii) describe the lived shared experiences of caregivers living with HIV-positive children.

## Data Analysis

In light of the exploratory nature and aims of this study, the data were analyzed through the process of IPA using NVivo8 software (15). Data analysis was approached as an inductive and iterative process consistent with IPA, in which all the respondents’ narratives were allowed to emerge (15). In keeping with IPA (15), the following steps were taken to analyze the data:

(1) In the first level of analysis, the transcripts of all the interviews were examined independently and subjected to open coding by means of making notes describing striking issues emerging from the narratives that allowed the researcher to study each transcript distinctly, in addition to comparing and contrasting varying responses as they emerged from each transcript to maintain the rigor inherent to IPA (15).
(2) Each key concept that emerged was broken into categories to provide meaning to the different aspects of a respondent’s experience of the phenomena under research (e.g., caregiving in the context of HIV). Each category of meaning was given a code that described the emergent meaning as reflected in the respondent’s words, such as associated health challenges. Once the entire transcript had been coded in this way, themes were extracted and listed. At this point, comparing data across categories, as well as across and between individual respondent, transcripts were facilitated to validate core categories and enhance the reliability of the data.
(3) The researcher looked for connections between and among themes in order to cluster them in a meaningful way. As a result, the researcher was able to identify umbrella, or superordinate, themes by linking all the subthemes (made up of a cluster of themes) that emerged, and then extracted them across all transcripts (16).
(4) Thereafter, an independent investigator (supervisor) was consulted to review subthemes and superordinate themes, with the identified themes being further saturated to improve validity. This process saw the supervisor independently assist with the verification and confirmation of themes. The process proved invaluable and pertinent, as the supervisor utilized it to subject the transcript to scrutiny and to observe the quality of the data collected. As indicated by Reid et al. (15), an independent audit of analysis was not only acceptable but also pivotal to the cross-validation of data which is consistent with IPA.

## Findings

The overarching themes that emerged were categorized into (1) contextual challenges impacting on caregiving in the context of HIV; (2) impact on psychological and social functioning of caregivers; and (3) coping strategies.

## Contextual Challenges Impacting on Caregiving in the Context of HIV

The findings showed that caregiving happens, not only in the context of maternal HIV infection but also in the contexts of inadequate material and financial resources, and single-head households where women assume the primary caregiving role (17). Caregiving is also complicated by the issue of maternal death and abandonment, where relatives (mainly grandparents) assume the role of primary caregivers of children infected with HIV (18).

### Unfavorable Physical Environment

#### Poor Quality Social Conditions

Poor quality social conditions was the lived reality in which caregiving was performed. Lack of structural resources which created an unfavorable living environment for the families was a major challenge. Living and raising a child in a shack or a hut was most common.

Respondent: And the house we live in has a leaky roof.

While another said:

Respondent: We live in a hut in the village and it leaks when it rains, no tap for water and toilet.

#### Overcrowding (Density)

As a result, overcrowding (density) was not uncommon as the women reported that they often had share living space and beds.

Respondent: It’s me, my grandmother and grandfather, two uncles, my sister, and my two children, and my uncle’s child.

Respondent: There’re 10 of us (in one shack).

#### Unemployment

Unemployment was attributed as one of the major factors contributing to this problem. The majority of these caregivers and family members were not formally educated and as a result not employable for high-income professions. For those caregivers who had a job, caring for their ill child took priority over employment, in spite of the knowledge that they might struggle to cope financially. The following excerpt reflects the views of most caregivers.

Respondent: That cost me my job because after she was with me, when I’d gone to work and had left her with someone, I’d come back and she’d have diarrhea. Which caused frequent trips to hospital; I decided if I want her to pull through I’ll have to stay at home.

#### Lack of Financial Resources and Support

As a result, lack of financial resources and support was something that caregivers had confronted on a daily basis. Lack of financial resources acted as a major socioeconomic stressor and exacerbated the already negative effects of HIV on both the caregiver and the child.

Respondent: It has affected it (lack of financial support affects their daily quality of living), sometimes it’s the middle of the month and there’s no food at home. The money’s finished. And you think that the children need to eat and she has lots of energy and will want to eat.

Most caregivers were dependent on a social grant to meet the basic needs of their ill children. In the absence of adequate financial support most families struggled with making ends meet. They often prioritized providing for the whole family; such as buying food, electricity, taxi-fare or fuel, etc., above that of just the child.

Respondent: Throughout the month I have to provide food from the little R280.

Respondent: When I have to come to check-ups I have no money for taxi to come. It’s difficult.

#### Lack of Nutrition

Lack of nutritional provision was reported by the caregivers. Most of the caregivers were unable to adequately meet the nutritional needs of the children due to the lack of resources available to them. This remained a serious challenge especially as the children and some of their caregivers were on ARVs or other medication that should have been taken with food.

Respondent: In the middle of the month and there’s no food at home.

Respondent: I’ve been told here in the hospital to get special foods for her. But we get village food. And things don’t go as planned.

Human immunodeficiency virus in families places additional strain on already limited human and economic resources (19). The finding supports the view that extended families assume caregiving responsibilities, especially grandparents (20) and that the process is difficult, especially because it is most often done in the context of physical and financial limitations (17, 21). The role of poverty in the study, like elsewhere, is showing that lack of financial resources predispose or creates the vulnerability among caregivers of HIV-positive children to be less involved, available and unable to adequately meet the basic needs of the children (22).

### Associated Health Challenges

#### Continuing Child Health Problems

Continuing child health problems were reported as a common feature by the caregivers. While it is common practice now for all children to have access to combination antiretroviral therapy (23), common infections and associated illness confronted infected children. The panic associated with every time the child gets sick combined with other stressors such as limited resources can exacerbate the negative consequences of HIV on the health and well-being of the child and even the caregivers. Child illness emerged as extremely burdensome, anxiety provoking, and stressful to caregivers.

Respondent: When he starts coughing I don’t waste time for it to get worse.

Respondent: Every now and then. She had to go every other day to hospital and you know the transport costs attached to that, and I worry because I don’t have the money to take her, so we end up walking long distance to the hospital and it makes me tired.

#### Caregiver Health Problems

Caregiver health problems were also reported by the caregivers. About half of the sample of caregivers interviewed were biological mothers who themselves were HIV+. Apart from HIV, some women reported additional challenges. One caregiver spoke about her mental illness and alcoholism.

Respondent: Because of a mental illness [I] took amitriptyline treatment but (was) not taking it properly too…Yes, even though on amitriptyline I was still drinking.

Also, another caregiver’s story captured the entrenched dilemma of having to care for herself and her child.

Respondent: When I think about it, I just think if only I was the only one sick. Because, for example, I’m on TB treatment (and) taking ARVs. Sometimes I forget to give (him) his treatment.

#### Relatives and Other Children with Health Problems

Even for the caregivers who were not HIV+ (non-biological mothers) having a lifestyle-related chronic illness was not uncommon, especially as majority of them were grandmothers. It was generally expected of them to assume the role of primary caregiver to all household members, irrespective of their own health status.

Respondent: I am already on Diabetes medicine and used to pills, we both had to eat pills only difference is I’m old and have no qualms about death but he is still a baby.

Respondent: My husband is also sick, he gets seizures, and he’s on medicine as well.

Respondent: Yes, [the] last born has Down syndrome. She is 14 and is a girl.

The social context of caregivers of HIV+ children were grounded in a reality confronting daily stressors associated with the disease and other related health threats (17, 24). The roles of caregivers showed that not only did they care for HIV-infected children but also had to care for relatives with other illness apart from their own (25).

## Impact of HIV on the Psychological and Social Functioning of Caregiver

### Caregiver Psychological and Emotional Function

Caregivers also reported fatigue associated with caring for the HIV-infected child but felt they had to prioritize the needs of the children above their own.

Respondent: You feel at times that I’m just tired and no energy to do anything…but I have to provide for them [the children] I can’t disappoint my children.

Feeling helpless and hopeless was also troubling.

Respondent: Since I’m facing this alone, there are days where you feel like you are stuck and don’t know what to do.

Caregivers also reported struggling with symptoms such as sad mood, lack of appetite, and lack of energy, which impaired their capacity to respond effectively to their children’s needs.

Respondent: sometimes my head feels hot, and feel so tired I just skip supper…it’s hard for me to eat, I had no appetite. It all on me…has to take care of a sick child.

Several studies exploring the psychological impact of HIV on caregiving echoed the same emerging narratives of the women from this rural community (24, 25). The importance of this finding is that it highlights that both biological and non-biological caregivers are vulnerable to developing emotional problems associated with depression. Caregivers of PHIV-infected children struggle with symptoms of depression that often leaves them feeling emotionally stressed and fatigued, especially given their the contextual realities, with research showing that some struggling with guilt associated with having passed on the virus to their children (in the case of biological caregivers) (22). Depression may limit the caregiver’s capacity to engage in a meaningful way with their children (26, 27) and thereby impacting negatively on their children’s developmental outcomes (28).

### Function of Stigma (Real or Imagined)

#### Paradox of Disclosure

Uncertainty, the fear of stigma and discrimination permeated caregivers’ realities and affected their decision-making ability to disclose or not to disclose their own status (in case of biological caregivers) or that of their children. This was accompanied by fear and anxiety, which entrapped these caregivers in social isolation.

Respondent: I’m not someone who likes to go to other people’s houses, I just stay at home because people judge you for being HIV positive and I’m avoiding that.

#### Secrecy/Isolation

Secrecy/isolation, due to the stigma surrounding HIV, prevented some caregivers from adequately caring for their children. Secrecy regarding a child’s status denied caregivers the opportunity to access adequate healthcare and family support (29), which impacted negatively on the child’s health.

Respondent: When she was sick often, she wasn’t being fed her medicine properly. Even the TB medicine she had; which the mother never said were for TB but from my experience I know what they were. The mother didn’t give her properly. She was neglecting to feed the baby medicine. Because she’d placed them in the wardrobe in an attempt to hide them from me…That’s when I heard the baby is positive and became sure it was true and looked after her.

For some, not disclosing is part of maintaining the secrecy to avoid stigma not only to self but also to protect the child. However, not disclosing the HIV diagnosis and maintaining secrecy had the potential of denying the caregivers of social support.

Respondent: I was afraid people not going to like my child now because of the HIV. So I tell myself going to keep this a secret.

For some women in the study who decided to disclose, vulnerability to being rejected was a possible consequence.

Respondent: The father and mother they were living together. There were mishaps after the positive test. They had lived peacefully before Thando (pseudo name) was born. Once he (husband) found out, he stops supporting.

Most of the studies showed that stigma adds to the already burdensome roles that caregivers of HIV-infected children had to assume (17, 22). Like the findings suggest isolation, silence, shame, etc., often prevents caregivers from accessing resources and social support that might have alleviated some of their stress associated with their unmet needs (28).

### Care Burden

Caregivers are often required to manage their own health, that of their ill child, and that of the family (24). Frequent medical appointments, unexpected hospitalizations, in addition to the day-to-day caregiving needs of the children were reported to be a huge burden to caregivers.

Respondent: It becomes especially difficult if you’re working, because you will have to leave work at any time due to phone calls about the baby being sick.

Respondent: There’s a lot that happens, because you have to look after your child and yourself. It’s harder if you have other children. Then if the baby gets sick and gets admitted into hospital, you have to take care of the other children, as well as working. After she gets out you have to be watchful.

While these caregivers understood their role as caregivers to their children, they nevertheless felt that their capacity to be effective parents was challenged as a direct result of the physical demands of caring for a child with HIV.

Respondent: Especially when she is being disobedient, and I ask myself why do I bother wasting my time raising her.

Respondent: There are days where you feel like you’re stuck and don’t know what to do. And you feel like you’re in way over your head and can’t cope. Especially if you’re alone…you just have no energy for anything or anyone…you feel like a failure.

Biological and non-biological caregivers (many having other chronic conditions) also experienced an ongoing struggle related to the balancing of their own health concerns with the health demands of their child.

Respondent: When I think about it, I just think if I only. If I was the only one sick. Not the child as well. Because for example I’m on TB treatment, taking ARVs, sometimes I forget to give him his treatment. And sometimes I’m in bed feeling awful and I have to get up and take care of him.

In the extract below from a biological parent, we see how guilt is twofold; on the one hand, they feel guilty about the child being infected because of them, and on the other hand, they feel guilty about not being able to adequately care for the child due to their own physical condition.

Respondent: When I think about it, I just think if I was the only one sick…and wish the situation changes and she becomes negative…it’s better if you are positive with no children who are positive. Then you don’t suffer…sometimes I’m in bed feeling awful and I have to get up and take care of him.

Caring for an HIV-infected child is difficult and it is a burden that associated with uncertainty, physically demanding, time consuming, and stressful (24). The burden was increased for biological caregivers who often had to confront challenging emotions of self-blame and guilt. Similarly, single caregivers (which was the case for most of the participants) already confronting financial challenges due to associated poverty (17, 24) experienced increase burden.

## Coping

### Negative Coping

#### Concealment of Health Status As a Coping Strategy

Biological mothers often utilized concealment as a means of coping with the illness. Fear and social rejection were some of the reasons mentioned for keeping the diagnosis secret. Concealment as a coping strategy had severe negative repercussions for the health of the children and their biological mothers. As reported by a non-biological caregiver, this had prevented her child from previously getting appropriate treatment and support.

Respondent: She was scared of me and didn’t tell me…she evaded the question and I left it at that. Then the baby got sick again. She has all the medicine hidden on top of the wardrobe. I tried to read it [the folder]. She’d take it from me, and tell me to disregard it.

### Positive Coping

#### HIV-Positive Child As a Source of Inspiration

Some biological caregivers find caring for their HIV-positive child a source of inspiration for negotiating with their inner fears, especially those associated with their own illness and having a future.

Respondent: There’s no way he won’t make it. Not while I’m still alive…what keeps me tough…the thing that keeps me going is the fact that I want to see him when he’s grown up. Everything I do I do for him.

#### Faith in God As a Source of Support

Drawing strength from their faith was essential for some caregivers to accept and cope with the child’s diagnosis of HIV.

Respondent: she was brought to me to raise, the first thing I did before I took the child. I knew her mother had died of AIDS and when she arrived I locked myself in my room and prayed so that I could accept the child and love her unconditionally. God gave me strength and I never had any problems and I was able to accept them into my heart.

#### Family and Friends As Sources of Support

Despite the fear associated with disclosure and the stigma attached to HIV, some of the caregivers were able to reach out for support. From their stories it was clear that disclosure did not always result in negative consequences, such as rejection and discrimination, but also provided the opportunity to give and receive support.

Respondent: …when I found out he is positive I had to tell her [mother] because she spends a lot of time with him [HIV positive child]…she had no problem with the news, and told me “let him stay with me and I’ll feed him his medicine”…

#### Health Staff As a Source of Support

For other caregivers, the help and proper information exchange from their healthcare providers assisted with acceptance and coping.

Respondent: The thing that helped me a lot was being counseled here in the hospital…was fine after that.

Respondent: now they explain things in detail to you…you have to accept your situation and understand…

The caregivers’ ability to appraise their stressful reality of caring for an HIV-positive child is an important predicting factor to the psychological outcome of both caregivers and their children (19). Consistent with the findings, in the review of existing research, Klunklin and Harrigan (17) found that “resilience, spirituality, and a commitment to kin in coping with AIDS” was very much central to caregivers of HIV-infected children (p. 291). Reliance on faith and spirituality emerged as an important symbol of the inner belief system that allowed the caregivers to draw strength despite the continuing daily adversities associated with HIV illness and contextual challenges (19, 24). In addition, the findings corroborated the supportive relationships provided by extended family, friends, and health professionals (19).

## Conclusion

This study is one of few that qualitatively explore the effects of HIV/AIDS on caregiving in a rural setting from a socio-ecological perspective, while trying to understand the systemic impact of the HIV/AIDS disease on the families. It reiterates the special role that caregivers play in the lives of HIV-infected children and the contextual challenges associated with caregiving in the context of HIV/AIDS within a resource constraint setting.

In sum, the study has assisted in identifying a number of issues that are pertinent to caregivers of HIV-infected children that provides new insight to the HIV/AIDS literature, which include that HIV/AIDS has a definitive systematic impact and to continue to view it as a disease that affects the individual solely is highly problematic and misguided. To view it systemically allows us to understand that caregiving is influenced by the broader socio-ecological risk factors such as poverty, caregiver mental health (30), health status of the child, and lack of social support (29, 31). Therefore, any intervention would benefit from incorporating these cumulative socio-ecological risk factors that families living with HIV/AIDS have to confront, rather than to focus just on an individualized medical management model of the disease. An intervention that is integrated within the broader socio-ecological context may be more effective at addressing the socioeconomic challenges and mental health problems associated with families living with HIV that seem to influence parenting and child developmental outcomes (32).

Limitations must be considered when interpreting the results. First, the sample is a convenience sample, recruited from an HIV clinic from within a general hospital that may not reflect the larger population of caregivers of urban setting in South Africa, particularly those not followed up in HIV care which speaks to the generalizability of the findings. Second, the study was cross-sectional and qualitative in nature and therefore precludes drawing any conclusions based on causality. Additional research is necessary to fully understand the complexity of caregiving in the context of HIV/AIDS and how the socio-ecological risk factors influence this process. Furthermore, future research on caregiving and HIV/AIDS should explore the role of social support.

## Ethics Statement

The study was approved by The University of KwaZulu Natal’s Biomedical Research Ethics Committee (Protocol Number: BE252/11) and Ethics Committee of East London Hospital Complex. To ensure confidentiality and anonymity of the participants, the names and location were not reported, as this could lead to identification. Informed consent was obtained from all participants.

## Author Contributions

The author confirms being the sole contributor of this work and approved it for publication.

## Conflict of Interest Statement

The author declares that the research was conducted in the absence of any commercial or financial relationships that could be construed as a potential conflict of interest.
